# Quantitative ultrasound delta-radiomics during radiotherapy for monitoring treatment responses in head and neck malignancies

**DOI:** 10.2144/fsoa-2020-0073

**Published:** 2020-09-04

**Authors:** William T Tran, Harini Suraweera, Karina Quiaoit, Daniel DiCenzo, Kashuf Fatima, Deok Jang, Divya Bhardwaj, Christopher Kolios, Irene Karam, Ian Poon, Lakshmanan Sannachi, Mehrdad Gangeh, Ali Sadeghi-Naini, Archya Dasgupta, Gregory J Czarnota

**Affiliations:** 1 Department of Radiation Oncology, Sunnybrook Health Sciences Centre, Toronto M4N 3M5, Canada; 2 Department of Radiation Oncology, University of Toronto, Toronto M5T 1P5, Canada; 3 Evaluative Clinical Sciences Platform, Sunnybrook Research Institute, Toronto M4N 3M5, Canada; 4 Department of Radiotherapy & Oncology, Sheffield Hallam University, Sheffield, UK; 5 Department of Physics, Ryerson University, Toronto M5B 2K3, Canada; 6 Department of Electrical Engineering & Computer Sciences, Lassonde School of Engineering, York University, Toronto M3J 1P3, Canada; 7 Department of Medical Biophysics, University of Toronto, Toronto M5G 1L7, Canada; 8 Physical Sciences Platform, Sunnybrook Research Institute, Toronto M4N 3M5, Canada

**Keywords:** biomarker, delta-radiomics, head and neck cancer, imaging, machine Learning, quantitative ultrasound, radiomics, radiotherapy, response, texture

## Abstract

**Aim:**

We investigated quantitative ultrasound (QUS) in patients with node-positive head and neck malignancies for monitoring responses to radical radiotherapy (RT).

**Materials & methods:**

QUS spectral and texture parameters were acquired from metastatic lymph nodes 24 h, 1 and 4 weeks after starting RT. K-nearest neighbor and naive-Bayes machine-learning classifiers were used to build prediction models for each time point. Response was detected after 3 months of RT, and patients were classified into complete and partial responders.

**Results:**

Single-feature naive-Bayes classification performed best with a prediction accuracy of 80, 86 and 85% at 24 h, week 1 and 4, respectively.

**Conclusion:**

QUS-radiomics can predict RT response at 3 months as early as 24 h with reasonable accuracy, which further improves into 1 week of treatment.

## Lay abstract

Patients with head and neck cancer are often treated with radiation, which usually spans over 6–7 weeks. The response is usually measured 3 months after treatment completion. In this study, we had performed ultrasound scans from the patient’s neck node during radiation treatment (after 24 h, 1 and 4 weeks). Artificial intelligence was used to interpret the ultrasound imaging and predict the response to radiation at the end of 3 months. The scans obtained after the first week were able to predict the treatment response with reasonable accuracy (86%).

Head and neck (H&N) cancers are a group of malignancies involving sites like the pharynx, larynx, oral cavity, nasal cavity, salivary glands and paranasal sinuses [1]. H&N cancers are the sixth most common cancers worldwide and are attributed to 550,000 new cancer diagnoses and 300,000 deaths annually [2]. The majority of H&N malignancies arise from the epithelial lining of the upper aerodigestive system, with 80–90% being squamous cell carcinomas. Treatment outcomes are largely dependent on the stage of disease at the time of presentation, etiopathogenesis and other factors such as smoking, human papillomavirus, patient performance status and compliance to treatment. Approximately 60% of patients are diagnosed with advanced-stage disease, often with metastatic regional lymph node (LN) involvement [1,3]. Radical radiotherapy (RT) comprises the standard of care for a significant group of patients with head and neck squamous cell carcinoma combined with concurrent systemic therapy (chemotherapy/targeted therapy) and results in excellent organ preservation rates [4,5]. Surgery forms the primary treatment for patients with malignancies involving the oral cavity or advanced pharyngeal/laryngeal tumors with cartilage erosion, or for patients having residual disease postRT.

Response following RT is assessed with imaging and clinical examination 10–12 weeks following RT completion [6,7]. Tumor size change is often a delayed manifestation resulting from the accumulation of cell death and microstructural changes within the treated tumor [8]. Monitoring treatment response at an early stage during treatment is of clinical interest to enable response-guided personalized therapy and consequently to improve survival outcomes and to decrease treatment-related toxicities. In recent years, advanced imaging analysis has been made possible with the introduction of computer vision and machine-learning algorithms, commonly recognized as the field of ‘radiomics’. Artificial intelligence helps interpret complex data and correlation with clinical end points. Computational analysis of imaging leads to the generation of multidimensional complex features from which simplified models are developed using machine-learning-based classifiers. In the study here, K-nearest neighbor (K-NN) and naive-Bayes algorithms were used for classification, using clinical treatment response as ground truth labels. A K-NN classifier can perform classification by determining the majority class of the *K-*NNs to an unlabeled data point, where K is a positive integer. The K-NNs are determined by computing the distance between the unlabeled data point and its neighbors. Similarly, a naive-Bayes methodology can be used to classify data using Bayes’ theorem. This classifier assumes that all of the input features are independent of one another.

For H&N malignancies, several studies have demonstrated the efficacy of radiomic analysis for different imaging modalities like computed tomography (CT), MRI and PET in predicting outcomes [9,10]. Quantitative ultrasound (QUS) can detect changes in tumor microstructure during treatment. These changes reflect variations in acoustic properties related to ongoing cell death *via* apoptosis as well as other changes in the tissue microstructure [11,12]. Clinical studies in patients with locally advanced breast cancer (LABC) have demonstrated that changes in QUS parameters early into neoadjuvant chemotherapy (NAC) can be used to predict treatment response [13–15]. Being an inexpensive, nonionizing and portable imaging modality, QUS has the potential to be used for the early detection of H&N tumor response to treatment. In this report, we present the results from the first clinical study investigating the role of QUS imaging data obtained during radical RT for patients with H&N malignancies in predicting long-term tumor response early after the start of RT.

## Materials & methods

### Patients

The human study protocol employed in this research was approved by the institutional research ethics board at Sunnybrook Health Sciences Centre, Toronto, Canada (ClinicalTrials.gov Identifier NCT03908684). After obtaining written informed consent, patients with a confirmed diagnosis of H&N carcinoma at their primary disease site or neck nodes (for carcinoma with unknown primary) were recruited for study participation. Specialist H&N pathologists tested for histological confirmation of disease, and additional tests were done to determine human papillomavirus or Epstein–Barr virus involvement [16–18], as part of the standard of care following institutional guidelines [19]. None of the patients included in the current analysis exhibited nonresponse to treatment. No patients had stable disease or progressive disease (nonresponder) within the first 3 months of treatment completion.

As part of the patients’ diagnostic workup, a pretreatment CT and MRI were completed for disease staging (along with PET-CT in selected patients as indicated clinically), which provided information regarding LN involvement and size. Patient disease, treatment characteristics and clinical outcomes were obtained from a prospectively maintained database and through electronic medical records, treatment planning systems and imaging. All patients were treated with radical RT using conformal image-guided techniques (intensity modulated RT) as per institutional practice, with 70 Gy/33 fractions delivered over 7 weeks to high-risk target volumes (primary and nodes). The use of concurrent systemic therapy was at the discretion of the responsible medical oncologist according to standard institutional practice.

### Treatment response evaluation

As part of the patients’ post-treatment response evaluation, MRI (with or without CT/PET-CT as decided by treating physicians) was acquired 3 months after the final dose of RT. Radiological end points were evaluated for the primary site and LN, based on standard response criteria in solid tumors (RECIST 1.1) [20]. Complete responders (CR) were defined as having a complete resolution of the primary tumor and for all pathologically enlarged LN measuring <10 mm (short axis), with others classified as partial responders (PR). None of the patients included in the analysis were nonresponders (either no response or progressive disease) during RT or at 3 months at the primary tumor site or associated nodes. Additional information, if available through histology or metabolic imaging, was used to supplement classification into CR or PR categories at 3 months.

### Ultrasound data acquisition

The treating radiation oncologist determined the index LN for individual patients as the largest or most prominent node clinically. An ultrasound scan of the target index LN was acquired immediately before starting RT and at 24 h, week 1 and 4 of patient treatment. Ultrasound radiofrequency (RF) data collection was performed by an experienced research sonographer using a custom-built ultrasound device (Elekta Ltd, Montreal, Canada) equipped with a linear 4D transducer (4DL14-5/38 Linear 4D, BK Ultrasound, MA, USA), which had a center frequency of approximately 8 MHz and a sampling rate of 40 MHz. Data were acquired along 256 lateral scan lines (3.8 cm lateral field of view) with a scan depth of 5 cm and focus depth of 2.5 cm. The transducer was focused on the midline of the enlarged LN, and RF data were acquired across the entire LN volume.

### QUS features

QUS spectral analysis was completed over a region of interest, spanning the entire volume of the target LN. Spatial parametric images were generated for each QUS parameter by applying a sliding window analysis technique with a 2 × 2 mm sliding window and a 94% overlap between the adjacent windows in both the axial and lateral directions.

Seven QUS parameters were computed using spectral analysis: mid-band fit, spectral slope (SS), spectral intercept (SI), attenuation coefficient estimate (ACE), spacing among scatterers (SAS), average scatterer diameter (ASD) and average acoustic concentration (AAC). In order to nullify the effects of system transfer functions and beamforming by the transducer, normalization was performed using reference data obtained with the same patient scan settings from a tissue-mimicking phantom. The phantom was comprised of 5–30 μm glass beads enclosed in a homogeneous medium of microscopic oil droplets, which were immersed in gelatin with an attenuation coefficient of 0.576 dB/MHz/cm and a speed of sound of 1540 m/s (Department of Medical Physics, University of Wisconsin, WI, USA). The mid-band fit, SS and SI parameters were derived by performing a linear regression over the mean log-compressed, attenuation-compensated, normalized power spectrum [21]. Attenuation correction was achieved using the point-compensation method [22] by estimating the ACE, adopting the reference phantom technique [23]. The ASD and AAC parameters were derived by fitting a spherical Gaussian form factor model to the backscatter coefficient computed using an attenuation-corrected normalized power spectrum. The SAS parameter was obtained by applying an autoregressive model onto the power spectrum of the sample, where the model parameters were computed employing the Burg’s recursive algorithm [24].

The mean values of QUS parameters were determined by averaging QUS parametric image pixel values.

### Texture features

Second-order statistical analyses were performed on QUS parametric maps using a grey-level co-occurrence (GLCM) technique [25], which represents the angular relationship and distances between neighboring pixels in QUS parametric maps. Further details and the interpretation of various GLCM features have been discussed in previous studies [25,26]. For each QUS parametric map, 16 GLCMs were constructed at four interpixel distances (1, 2, 3 and 4 pixels) and four directions (0, 45, 90 and 135°). Four textural features were determined from each QUS parametric map and were comprised of GLCM contrast (CON), correlation (COR), energy (ENE) and homogeneity (HOM) values.

At each experimental assessment time, seven QUS and 24 texture features were obtained (texture analysis was not performed for ACE), leading to a total of 31 features. For analysis and development of the radiomics model, the changes in the values for individual features at the fixed scan assessment times (24 h, week 1 and 4) were computed by subtraction of the values obtained from baseline before starting RT. In the following sections, specific features are referred to as the differences from baseline rather than the absolute values, unless otherwise specified. As the values that were used in building the classifiers were the differences at various time points, the radiomics models used here were based on delta-radiomics.

### Statistical analysis

A Shapiro–Wilk test was conducted to determine the distribution of data for the different parameters. The normally distributed parameters were tested using an independent sample *t*-test (two sided, 95% CI) in order to investigate the differences between PR and CR. Other parameters were tested with the Mann–Whitney U-test (two sided, 95% CI). A Kaplan–Meier product-limit method was used to determine the survival analysis, and a log-rank test was conducted for univariate analysis. These statistical tests were performed on SPSS V.22 (IBM Corporation, NY, USA). The threshold for significance was set to p < 0.05.

### Classification modeling

K-NN and naive-Bayes algorithms were used for classification, using clinical treatment response as ground truth labels. Mean QUS and texture parameters were used as classifying features. In order to ensure optimal performance of the model and to prevent overfitting, only a few selected parameters were used as features in classification processes. In order to mitigate the curse of dimensionality, the maximum number of features used in the classification was set to three based on the rule of thumb (number of subjects/10). The best features were acquired through sequential forward selection in a wrapper framework. This method uses leave-one-out cross-validation to select features, which results in the highest prediction accuracy. Multivariable class analysis was also tested and compared with the single-feature model. Up to three of the best features were picked, and class analysis was performed. Based on the results, the performance of the classifier was evaluated by determining the sensitivity (%), specificity (%) and accuracy (% Acc). A receiver operator characteristic curve was generated, and the area under the curve was calculated. For the feature determination and machine-learning classifiers, MATLAB (R2016a, MathWorks, MA, USA) was used.

## Results

### Patient, disease & treatment characteristics

A total of 36 patients (33 males and three females) were included in this analysis. The median age was 60 years (range 40–82 years). As defined earlier, response assessment at 3 months following treatment completion was taken as an end point, which revealed 14 patients to be CR, and the remaining 22 to be PR. Details for clinical and treatment parameters are presented in Table 1. Concurrent chemotherapy was given along with RT in 30 (83%) patients, while one (3%) received cetuximab.

**Table 1.** Patient, disease and treatment characteristics for the study participants.

Patients and tumor characteristics n = 36 (all subjects)	n (%)
Age (years): – Median (range)	60 (40–82)
Sex: –Males –Females	33 (92) 3 (8)
Site: –Oropharynx –Larynx –Hypopharynx –Left parotid ––Nasopharynx –Carcinoma unknown primary	26 (72) 4 (11) 2 (6) 1 (3) 1 (3) 2 (5)
Human papillomavirus: –p16+ –p16- –Not specified/–unclear	24 (67) 1 (3) 11 (30)
**Stage**	
Primary tumor (T): –T0 –T1 –T2 –T3 –T4	2 (5) 9 (25) 15 (42) 5 (14) 5 (14)
Node involvement (N): –N1 –N2 –N3	21 (58) 10 (28) 5 (14)
**Systemic therapy**	
Chemotherapy: –Cisplatin (high and low dose) –Carboplatin –Carboplatin + etoposide –Cisplatin + carboplatin	25 (69) 1 (3) 2 (5) 2 (5)
EGFR inhibitor: –Cetuximab	1 (3)
None	5 (14)
**Response at 3 months**	
Treatment response classification: –CR –PR	14 (39) 22 (61)

CR: Complete responders; EBV+: Epstein–Barr virus-positive carcinoma; N: Nodal staging (AJCC 8th edition); p16+: Human papillomavirus-positive tumor; PR: Partial responders; T: Primary tumor staging (American Joint Committee on Cancer [AJCC] 8th edition).

### Clinical outcomes

**Figure 1.**
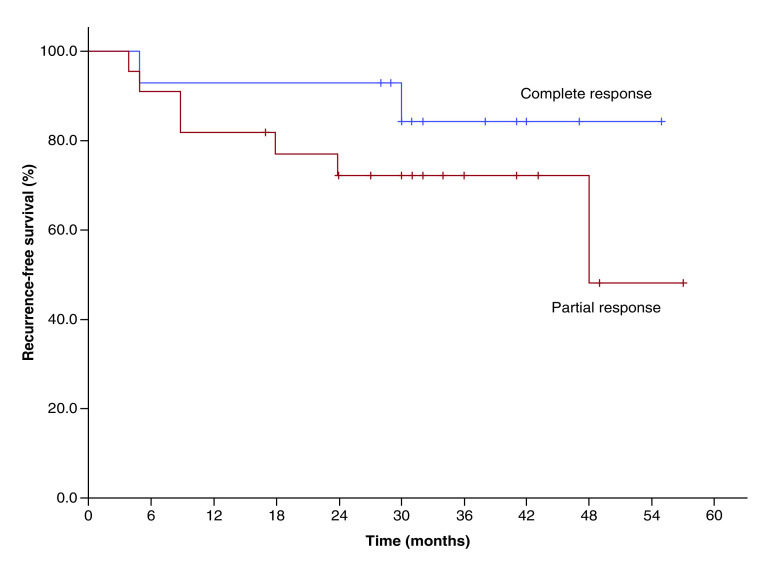
Kaplan–Meier survival plot showing recurrence-free survival for the complete responder and partial responder.

The median follow-up for all patients was 32 months (range 12–57 months). Disease recurrence was seen in nine patients (one had local recurrence, one had regional recurrence in neck nodes, others had distant metastases with or without locoregional relapse), of which two patients were from the CR group, and the remaining seven patients were from the PR group. Three-year recurrence-free survival for CR and PR patient groups was 84 and 72%, respectively (p = 0.042). Figure 1 displays the survival plots determined for the two groups.

### Feature analysis & classification results

**Figure 2.**
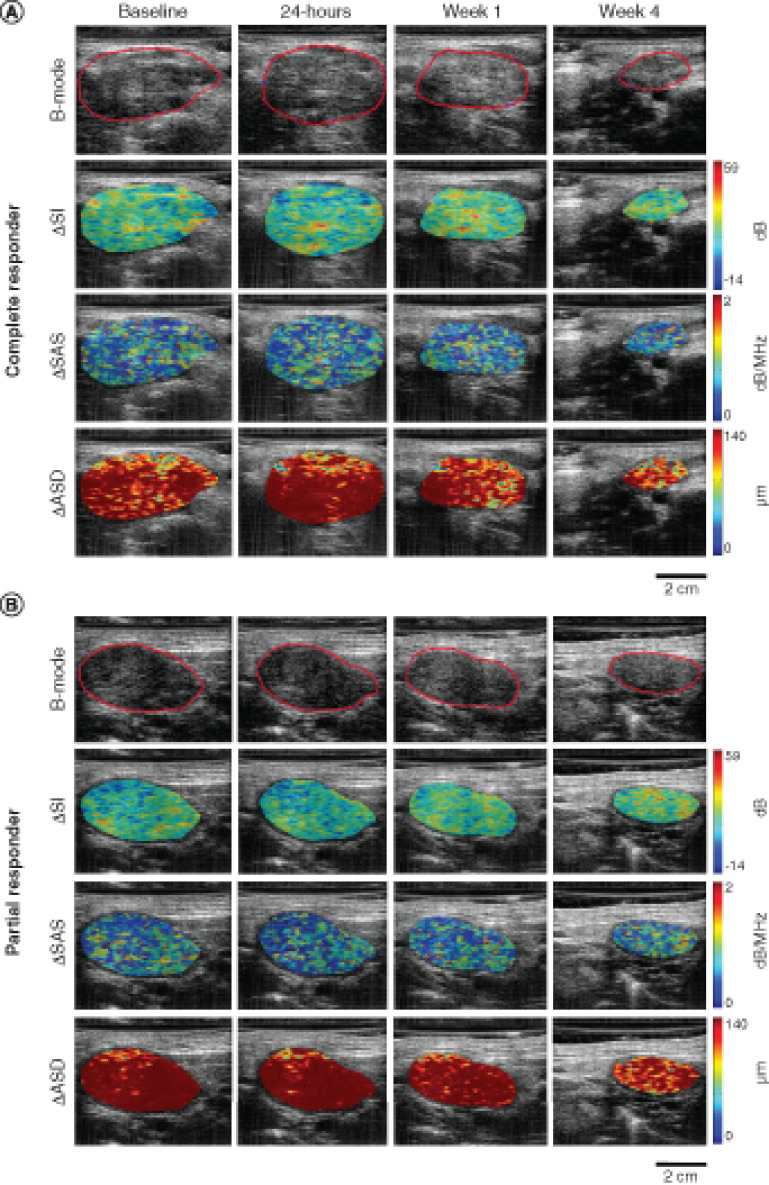
Quantitative ultrasound parametric maps. Representative QUS parametric image overlays of ΔSI, ΔSAS and ΔASD at baseline, 24 h, week 1 and 4 of treatment for a complete responder **(A)** and a partial responder **(B)**. The ultrasound B-mode images have been contoured to delineate the lymph node that was scanned. ASD: Average scatterer diameter; QUS: Quantitative ultrasound; SAS: Spacing among scatterers; SI: Spectral intercept.

Ultrasound B-mode images and corresponding SI, SAS and ASD parametric maps of patients from each of the response groups are presented in Figure 2. Two texture features at 24-h post-treatment, ΔSI-ENE and ΔSAS-HOM were found to be statistically different between CR and PR with p-values of 0.044 and 0.021, respectively, as noted in Table 2. One QUS texture parameter, ΔASD-COR, was trending toward significance (p = 0.056). Scatter plots of all the QUS mean value and textural parameters obtained for CR and PR at 24-h post the first fraction of RT are presented in Supplementary Figure 1. At other time points, no other features were found to have different distribution between the two groups.

**Table 2.** Twenty four-hours post-treatment quantitative ultrasound mean spectral and texture values for the most significant features demarcating complete responders from partial responders.

Parameter	p-value	CR (mean ± SEM)	PR (mean ± SEM)
Δ**SI-ENE**	**0.044**	**0.002 ± 0.001**	**-0.004 ± 0.030**
Δ**SAS-HOM**	**0.021**	**0.001 ± 0.002**	**-0.035 ± 0.030**
ΔASD-COR	0.056	0.010 ± 0.008	-0.047 ± 0.038

Bolded parameters demonstrate statistical significance. Other features approach near significance.

ASD: Average scatterer diameter; CR: Complete responders; COR: Correlation; ENE: Energy; HOM: Homogeneity; PR: Partial responders; SAS: Spacing among scatterers; SEM: Standard error of the mean; SI: Spectral intercept.

The classification results obtained using K-NN and naive-Bayes algorithms are displayed in Table 3. Overall, the univariate naive-Bayes model performed best in predicting treatment response at all assessment times. For the 24-h time point, the change in the AAC (ΔAAC-CON) feature demonstrated a classification Acc of 80%. Acc of 86 and 85% were demonstrated at week 1 and 4 of treatment for the change in SS (ΔSS-COR) and the change in ACE (ΔACE), respectively.

**Table 3.** Results for the best single-feature (A), two-feature (B) and three-feature (C) prediction models generated from machine-learning algorithms, K-nearest neighbor and naive-Bayes at 24-h post the first radiation treatment, week 1 and 4 of treatment.

A: single-feature classification						
Classifier model	Time point	%S_n_	%S_p_	AUC	%Acc	Best univariate feature
naive-Bayes	24 h	77	83	0.67	80	ΔAAC-CON
	Week 1	85	86	0.77	86	ΔSS-COR
	Week 4	84	85	0.79	85	ΔACE
K-NN	24 h	75	70	0.74	72	ΔAAC-CON
	Week 1	75	85	0.81	81	ΔSAS-ENE
	Week 4	76	79	0.80	77	ΔASD-ENE
**B: two-feature classification**						
**Classifier model**	**Time point**	**%S_n_**	**%S_p_**	**AUC**	**%Acc**	**Best two features**
naive-Bayes	24 h	67	73	0.64	70	ΔMBF + ΔAAC-CON
	Week 1	76	84	0.67	80	ΔSS + ΔAAC-COR
	Week 4	75	77	0.75	76	ΔACE + ΔASD
K-NN	24 h	74	78	0.78	76	ΔSS + ΔAAC-CON
	Week 1	73	78	0.77	76	ΔSS + ΔSAS-ENE
	Week 4	76	82	0.81	79	ΔSS-ENE + ΔASD-ENE
**C: three-feature classification**						
**Classifier model**	**Time point**	**%S_n_**	**%S_p_**	**AUC**	**%Acc**	**Best three features**
naive-Bayes	24 h	63	69	0.63	66	ΔMBF + ΔSAS-CON + ΔAAC-CON
	Week 1	68	78	0.65	73	ΔSS + ΔSS-COR + ΔAAC-ENE
	Week 4	66	64	0.61	65	ΔMBF + ΔACE + ΔASD
K-NN	24 h	71	76	0.76	77	ΔSS + ΔSI-ENE + ΔAAC-CON
	Week 1	73	81	0.75	77	ΔSS + ΔMBF-ENE + ΔSAS-ENE
	Week 4	79	80	0.82	80	ΔSS-ENE + ΔSI-ENE + ΔASD-ENE

AAC: Average acoustic concentration; Acc: Accuracy; ACE: Attenuation coefficient estimate; ASD: Average scatterer diameter; AUC: Area under curve; CON: Contrast; COR: Correlation; ENE: Energy; HOM: Homogeneity; K-NN: K-nearest neighbor; MBF: Mid-band fit; SAS: Spacing among scatterers; SI: Spectral intercept; S_n_: Sensitivity; S_p_: Specificity; SS: Spectral slope.

**Figure 3.**
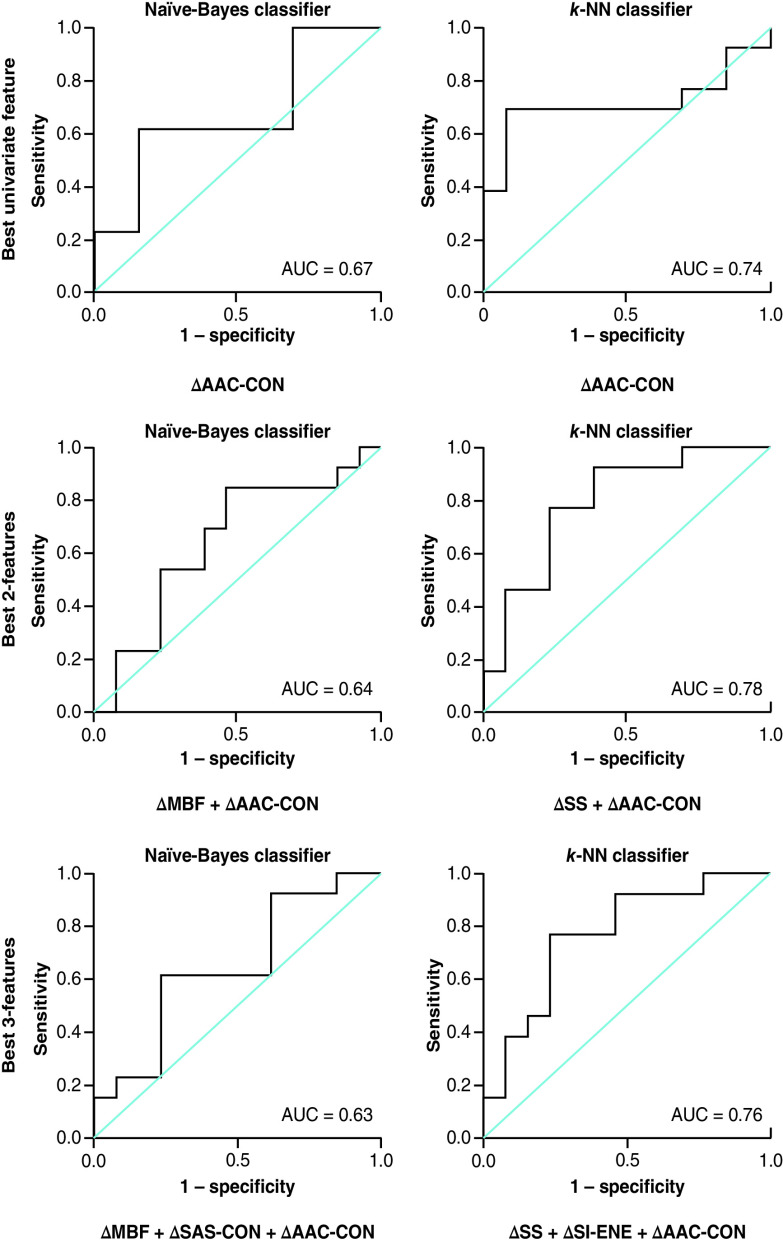
Results for the best single-, two- and three-feature classification using naive-Bayes and K-nearest neighbor classifier models at 24 h after the initial radiation therapy treatment (receiver operating characteristic curve presented). AUC: Area under the curve; CON: Contrast; ENE: Energy; K-NN: K-nearest neighbor; MBF: Mid-band fit; SAS: Spacing among scatterers; SI: Spectral intercept; SS: Spectral slope.

**Figure 4.**
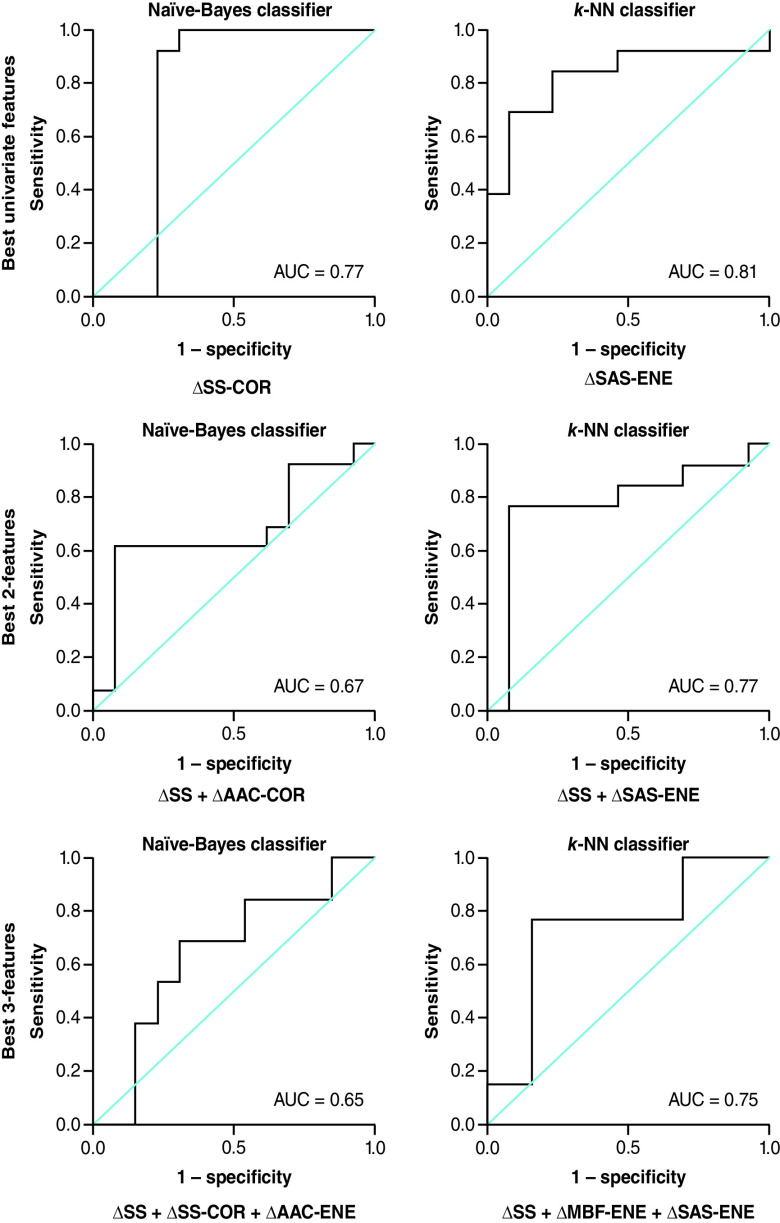
Results for the best single-, two- and three-feature classification using naive-Bayes and K-nearest neighbor classifier models at week 1 of radiation treatment (receiver operating characteristic curve presented). AUC: Area under the curve; CON: Contrast; COR: Correlation; ENE: Energy; K-NN: K-nearest neighbor; MBF: Mid-band fit; SAS: Spacing among scatterers.

**Figure 5.**
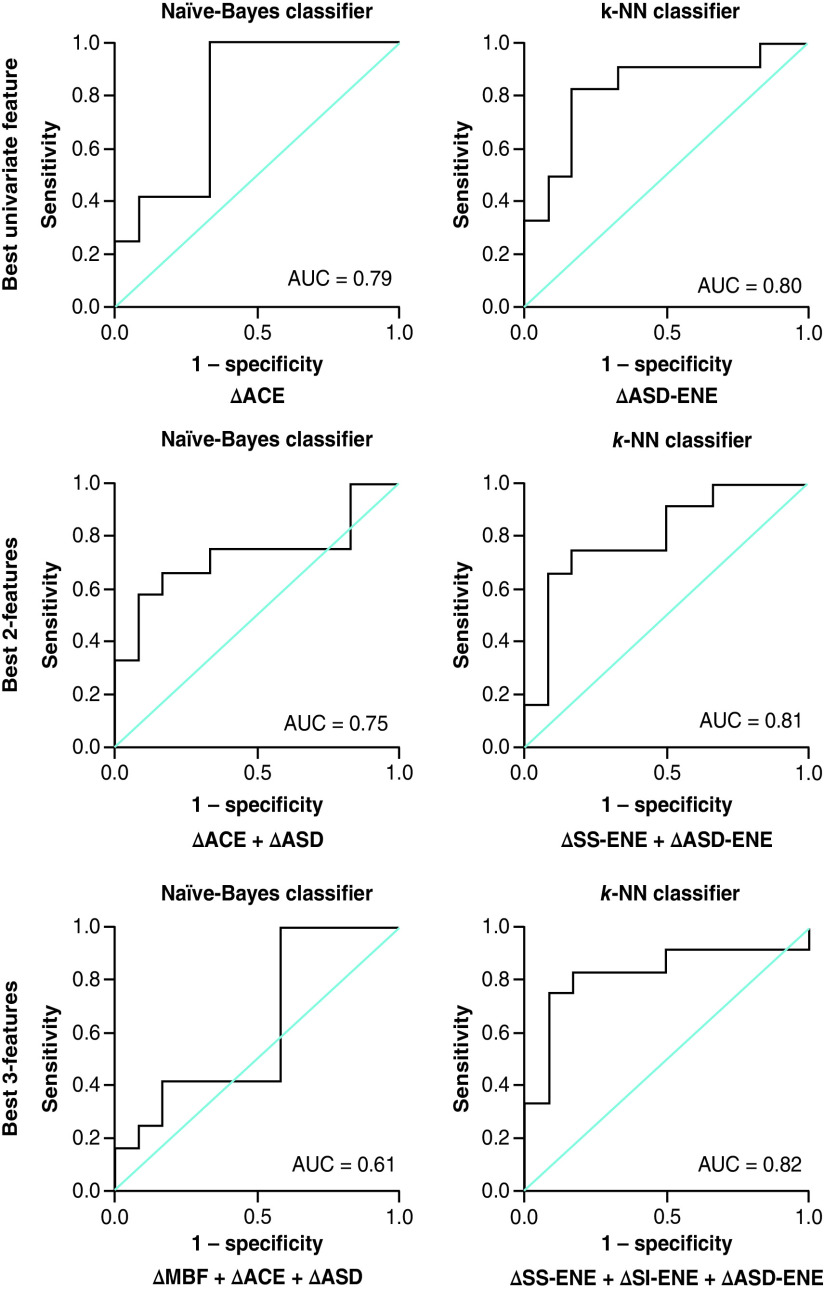
Results for the best single-, two- and three-feature classification using naive-Bayes and K-nearest neighbor classifier models at week 4 of radiation treatment (receiver operating characteristic curve presented). ACE: Attenuation coefficient estimate; ASD: Average scatterer diameter; AUC: Area under the curve; CON: Contrast; ENE: Energy; K-NN: K-nearest neighbor; MBF: Mid-band fit; SI: Spectral intercept.

For the K-NN classifier, multifeature models improved the classification Acc at week 4 of RT. For one-feature classification models, the change in ASD (ΔASD-ENE) resulted in a classification Acc of 77 %. With two features, the Acc increased to 79 % (ΔSS-ENE + ΔASD-ENE) and using three features (ΔSS-ENE + ΔSI-ENE + ΔASD-ENE) increased the Acc to 80 %. Figures 3, 4 & 5 represent the receiver operator characteristic curves for naive-Bayes and K-NN classification models using one, two and three features from QUS data acquired at 24 h, week 1 and 4 of treatment, respectively.

## Discussion

In the past several decades we have witnessed a paradigm shift in the management of H&N malignancies, with radical RT recognized as the standard of care along with concurrent chemotherapy for primary tumors of the pharynx and larynx. These have led to better organ preservation rates as critical functions like swallowing, speech and breathing are co-ordinated through H&N anatomical structures. The introduction of intensity-modulated RT and image guidance has further helped in the alleviation of normal tissue toxicities like xerostomia [27]. Despite these advancements, a significant proportion of patients develop disease recurrence, and most patients cured of the disease continue to suffer long-term treatment-related toxicities affecting their quality of life [28,29]. This has spurred the development of useful biomarkers that can be used to monitor cell death in real time while a patient is undergoing treatment and to predict their overall treatment response. Subsequent personalized risk-adapted treatments can lead to appropriate radiation dose escalation or de-escalation strategies designed to find the optimal balance between cure and toxicities based on an individualized approach. Our study is the first clinical report of using QUS delta-radiomics during RT, as a simple, rapid, inexpensive imaging modality to predict treatment response to radical RT for H&N malignancies. Delta-radiomics denote the changes of radiomic features over time associated with tumor changes resulting from treatment. Delta-radiomics determined from QUS imaging data has previously been demonstrated to be an effective strategy to study and predict the response to NAC in breast cancer [14].

Using naive-Bayes classification, single-feature selection performed best for all time points in this study compared with multiple feature selection. At 24 h after the start of radiation, the best feature was found to be the change in the CON of the AAC parametric map (ΔAAC-CON). This may be due to early changes in the concentration of scatters. At week 1, the change in the COR of the SS (ΔSS-COR) was the best feature. The SS is related to scatterer shape and size [11,30,31]. In a previous study, SS has also been found to closely correlate with cell death through the production of apoptotic bodies and nuclear fragmentation [32]. The change in SS may be due to cell fragmentation or changes in the structure of the cell’s nucleus. A preclinical study by Vlad *et al.* found that SS and ultrasound integrated backscatter parameters changed when tumors were exposed to radiation [33]. Four weeks after the start of RT, the best classifying feature was the change in the ACE (ΔACE). The attenuation coefficient is related to the composition and density of the tissue [34]. This feature has been used in other studies to differentiate fatty from healthy liver tissue and as a quantitative descriptor in breast masses [35,36].

Using the K-NN classifier, the single-feature analysis performed best with week 1 data (ΔSAS-ENE), while multifeature analysis performed best with data acquired at 24 h (ΔSS + ΔAAC-CON) and in week 4 (ΔSS-ENE + ΔASD-ENE) QUS data. The features that were selected mainly involved texture features derived from parametric maps related to scatterer size, concentration and scatterer spacing.

Statistical analysis of changes in the mean and texture parameters indicated that the ΔSI-ENE and ΔSAS-HOM at 24 h after the initial treatment were the only parameters that were found to be significantly different between CR and PR. The 24-h ΔSI-ENE parameter was greater for CR compared with PR. The SI parameter is related to the scatterer size and composition in tissue microstructure [37,38]. This suggests that there is more significant order in tissue structure of the LN 24 h after initial treatment for CR compared with PR. The PR demonstrated a lesser ΔSAS-HOM compared with CR. This may reflect less HOM in the spacing among the scatterers in the LN for PR.

The role of QUS in medicine and oncology is emerging with previous studies demonstrating QUS as an effective modality for the monitoring of treatment response in patients with LABC receiving NAC. Sannachi *et al.* found that a combination of mean QUS spectral, texture and molecular features was able to classify CR, PR and nonresponders at 1, 4 and 8 weeks into chemotherapy with Acc of 78, 86 and 83%, respectively [14]. That study found that changes in scatterer (lobule) spacing (SAS) occurred early on in week 1 after the start of NAC, and changes in the size of scatterers (lobules) occurred later in week 4. Tadayyon *et al.* also used pretreatment QUS data to predict LABC patient response to NAC with an Acc of 88 % [15]. QUS has also been shown to differentiate between benign and malignant tissue and to classify tumor grade with high Acc [39,40]. Furthermore, a previous study had shown that preradiation QUS data could predict response at 3 months with an Acc of 88% in H&N malignancies [41]. Although traditional B-mode ultrasound has been used in the assessment of morphological changes of neck nodes in H&N malignancies, the application of QUS can provide quantitative data to better estimate the ongoing treatment-related changes.

Radiomic analysis has been undertaken using other imaging modalities to assess treatment response and clinical outcomes for different H&N malignancies [42]. A study by Vallières *et al.* used PET-CT to evaluate risks for recurrence in H&N cancer using texture, shape, intensity and genomic features [10]. Dynamic contrast-enhanced MRI has been used to predict response in patients with H&N cancer. Cao *et al.* distinguished patients controlled at the primary site from ones having disease relapse by analyzing the tumor blood volume and flow pretreatment at 2 weeks into treatment [43]. In another study by King *et al.*, diffusion-weighted MRI (DW-MRI) was used to measure the apparent diffusion coefficient, which demonstrated a significant decrease of the apparent diffusion coefficient 2 weeks after the start of RT [44]. Imaging is crucial in the determination of treatment outcomes. Studies used DW-MRI and perfusion-weighted MRI to detect recurrent H&N cancer and to differentiate from postradiation changes [45–47]. PET-based response monitoring showed blood flow parameters to be accurate predictors of metabolic response [48]. Relative factors to consider for those imaging modalities include their cost, scan duration, radiation exposure (CT/PET), the need for contrast agents and related toxicities. Ultrasound has the benefit of being relatively low cost, does not emit ionizing radiation and does not require the administration of exogenous contrast agents resulting in potentially better patient compliance. This is the first radiomics study involving an RF-based modality like ultrasound using the change in QUS parameters during RT to predict treatment response in H&N patients. The work identifies that QUS is capable of detecting changes in tissue microstructure as early as 24 h into treatment. This motivates the use of ultrasound as a technique to monitor the LNs of H&N patients and as a method to evaluate treatment efficacy.

The imaging target in this study was the largest metastatic LN, which was used to demonstrate that changes in the biological structure of the LN following RT correlate with patient response to RT. LN response has been shown to correlate with locoregional control in patients receiving RT for H&N cancer [49,50]. Hauser *et al.* demonstrated that the DW-MRI parameter associated with the vascular and perfusion signal in metastatic H&N LNs was able to distinguish between patients with and without locoregional control after RT treatment [49]. Goguen *et al.* identified a correlation between positive LNs and decreased overall survival and progression-free survival in patients after receiving chemotherapy and RT [51]. In the study here, although a proportion of the patients were having relatively lesser follow-up, a significant difference of recurrence-free survival was seen between the CR and PR.

The advantage of QUS-radiomics is the development of biomarkers from a noninvasive imaging modality. Rapid-scan acquisition and excellent patient compliance make it an attractive strategy, as treatment response can be monitored in real time early in the course of treatment.

## Limitations

There are a few limitations to the work conducted here. First, the primary tumor site was not imaged as these are structures located at depths within the body that are not accessible by ultrasound imaging. Nonetheless, it is promising that features from bulky LNs by themselves can predict the overall response of both the primary disease and the disease in LNs. Additionally, given this was a pilot study to explore feasibility and acquire preliminary results, the number of patients included in this report has been relatively small. With promising initial results, the acquisition of additional data will lead to greater confidence in the interpretation of the results. Further expansion of the study cohort to include patients from other institutions, and independent external validation, will help in testing the utility of the current feature set with the development of a reproducible feature set. More recent work has examined interinstrument variability as well as interuser variability and found those sources of variance are not significant [52].

## Conclusion

QUS delta-radiomics has the potential to assess treatment response early in the course of therapy. QUS data obtained as early as 24 h after starting RT can predict response at 3 months with an Acc of 80%. Additional features obtained at week 1 and 4 of RT further improved the prediction rates to 85% approximately. Being a relatively inexpensive, portable and simple imaging modality, QUS-radiomic signatures should be further investigated as an option to monitor cell death and treatment response in patients with H&N malignancies treated with radical RT.

## Future perspective

Future developments could include incorporating QUS-radiomic prediction models in guiding treatment escalation and de-escalation based on real time response during RT. Additionally, classification performances can potentially be improved through additional imaging analysis accounting for changes in tumor shape, grey-level histograms and using other methods of texture analysis as well as integration with clinical and genomic markers [53]. Finally, once more data are acquired, more advanced machine-learning algorithms such as deep-learning methods, could be tested for their response assessment capability.

**Executive summary**

**Background**

- This study investigated the use of quantitative ultrasound (QUS) delta-radiomic biomarkers to predict treatment response in patients with head and neck (H&N) cancer treated with radical radiotherapy (RT).

**Materials & methods**

- Thirty six patients with node-positive H&N cancer underwent ultrasound imaging of metastatic lymph nodes at 24 h, 1 and 4 weeks after starting RT.
- QUS spectral and texture parameters were extracted from the ultrasound data.
- Machine-learning algorithms were used to develop response prediction models.

**Results**

- Using the naїve-Bayes classification algorithm, the response prediction accuracies were 80, 86 and 85% using QUS features acquired at 24 h, 1 and 4 weeks into RT, respectively.

**Conclusion**

- QUS delta-radiomics was used to predict treatment response to radical RT in H&N malignancies with reasonable accuracy. The best results were obtained after 1 week of treatment.
