# Supplementary figures and images for: Quantitative ultrasound delta-radiomics during radiotherapy for monitoring treatment responses in head and neck malignancies
Source: Future Sci OA. 2020 Sep 4;6(9):FSO624. doi: 10.2144/fsoa-2020-0073 (PMC7668124; doi:10.2144/fsoa-2020-0073)

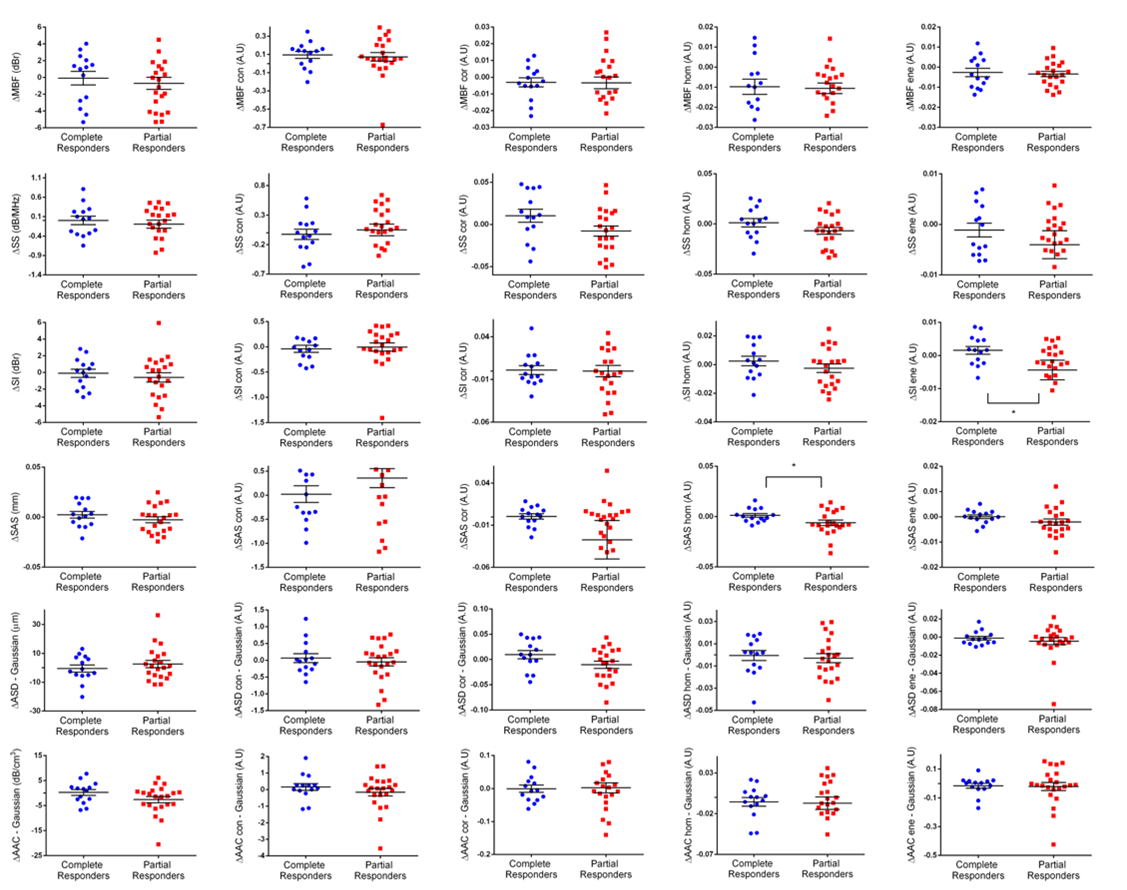

Supplement: Supplementary file 1 [file fsoa-06-624-s1.tif]
